# Gradient‐Oxidized Bacterial Cellulose for Self‐Sustained Soil Moisture Sensing

**DOI:** 10.1002/advs.78511

**Published:** 2026-10-27

**Authors:** Nan Zhou, Jing Li, Xiang Xu, Senyuan Ye, Bin Chen, Shuang Wu, Duo Xu, Keshuai Liu, Chong Gao, Weilin Xu, Can Ge

**Affiliations:** ^1^ State Key Laboratory of New Textile Materials and Advanced Processing Wuhan Textile University Wuhan China; ^2^ Department of Electronic Engineering and Shun Hing Institute of Advanced Engineering The Chinese University of Hong Kong, Sha Tin, New Territories Hong Kong China; ^3^ Hubei Institute of Aerospace Chemical Technology Xiangyang China; ^4^ College of Textile Science and Engineering Zhejiang Sci‐Tech University Hangzhou China; ^5^ National Key Laboratory of Aerospace Chemical Power Xiangyang China; ^6^ College of Textile and Clothing Engineering Soochow University Suzhou China

**Keywords:** bacterial cellulose, biodegradable electronics, hydrovoltaic effect, soil moisture sensing

## Abstract

Soil moisture monitoring is pivotal to precision agricultural irrigation. Conventional soil moisture sensors rely on external power supplies and suffer from poor environmental compatibility. The facile fabrication of hydrovoltaic devices with asymmetric film structures remains challenging, and related designs tailored for soil scenarios are still insufficient. Here, we report an in situ‐grown planar asymmetric encapsulated bacterial cellulose (AEBC) device for self‐powered soil moisture sensing. Graphene oxide (GO) and carbon nanotubes (CNT) are sequentially embedded on opposite sides of the growing cellulose network via gravity‐assisted in situ assembly, forming an oxygen‐functional gradient. A LiCl‐doped Ecoflex hygroscopic layer is further integrated to continuously provide mobile ions and stabilize moisture uptake. The 35 µm‐thick device delivers a volumetric power density of 211.37 µW·cm^−3^ and maintains stable operation for over 20 days across wide temperature and pH ranges. A wireless monitoring system integrating the AEBC with deep learning‐based signal recognition achieves accurate soil moisture detection. The bacterial cellulose matrix can be biodegraded in soil, and degradation residues exert no adverse effects on plant growth. This work provides an in situ fabrication strategy for asymmetric hydrovoltaic films, offering a promising sustainable platform for self‐powered agricultural monitoring.

## Graphical abstract

An asymmetric encapsulated bacterial cellulose device integrates a gradient‐oxidized BC framework with a LiCl‐doped Ecoflex hygroscopic layer, enabling self‐powered soil moisture monitoring through directional ion transport. The device sustains stable hydrovoltaic output, supports deep‐learning humidity classification, and fully biodegrades in soil.

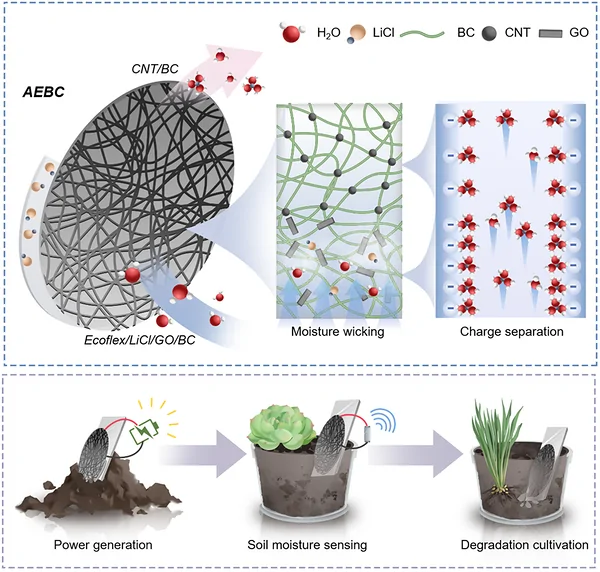

## 1 Introduction

Soil moisture is a critical parameter governing agricultural productivity and terrestrial ecosystem dynamics. In situ high‐resolution soil moisture monitoring is essential for precision irrigation and drought mitigation [1, 2]. However, existing sensing technologies struggle to simultaneously meet the requirements of accuracy, durability, and environmental friendliness [3, 4]. Conventional resistive/capacitive sensors suffer from limited sensitivity and are prone to electrode corrosion and salinity interference. Although frequency‐domain/time‐domain reflectometry techniques offer higher accuracy, their reliance on expensive instrumentation and external power supply restricts large‐scale deployment in off‐grid environments. In addition, the accumulation of non‐degradable electronic waste contradicts the goals of sustainable agriculture [5, 6, 7]. Therefore, developing self‐powered, environmentally friendly, and robust soil moisture sensors remains a critical challenge.

The hydrovoltaic effect has recently emerged as a promising mechanism for self‐powered sensing, which refers to the electrical potential generation through directional ion migration during the interaction between functional materials and water molecules [8, 9, 10]. Biomass bacterial cellulose (BC) has emerged as an ideal substrate for constructing moisture‐responsive devices due to its three‐dimensional nanofibrous network, abundant hydrophilic hydroxyl groups, and complete biodegradability [11, 12]. Carbon materials with rich oxygen functional groups can be composited with BC to form a hydrophilic network possessing a redox gradient, thereby significantly enhancing water molecule adsorption efficiency and charge separation capability [13, 14]. However, existing research on hydrovoltaic devices has primarily focused on optimizing output performance under ideal humidity conditions, and several critical deficiencies including thermodynamic instability, mechanical mismatch, and weak robustness remain regarding their applicability in real soil environments: (1) Effective hydrovoltaic output can be maintained for only several hours in open soil settings due to rapid dissipation of the ionic concentration gradient. (2) Mechanical mismatch between rigid structures and soil deformation leads to interfacial contact failure. (3) Chemical perturbations in open soil environments alter interfacial physicochemical properties and disrupt continuous ion transport pathways, thereby interfering with monitoring sensitivity [15, 16, 17, 18].

To address these challenges, we propose an asymmetric encapsulated BC (AEBC) with gradient oxidation and selective encapsulation. The BC nanofibrous substrate acts as a mechanically adaptive transport scaffold, conformally interfacing with deformable soil and preserving continuous water/ion transport pathways under dynamic conditions. An ionic gradient is established via rational decoration of carbon nanotubes (CNT), graphene (GO), and lithium chloride (LiCl). The hygroscopic LiCl enables continuous regeneration of ionic asymmetry within the built‐in carbonaceous electric field via moisture uptake and charge separation. This transforms the hydrovoltaic process from a transient response into a self‐sustained non‐equilibrium driving system. Selective Ecoflex encapsulation further serves as a mass transport regulator, suppressing ion loss and environmental interference while stabilizing internal gradients through controlled moisture flux and enhanced interfacial adhesion. Hence, AEBC delivers persistent energy output (211.37 µW/cm^3^) and reliable soil moisture sensing (accuracy of 98.6% across 10%–50% humidity), while its inherent biodegradability enables regenerative composting into soil systems. This work opens a new pathway for self‐powered, high‐sensitivity, and degradable moisture sensing technologies in complex soil environments.

## 2 Results and Discussion

### 2.1 Material Design and Characterization

AEBC consists of an integrated heterogeneous BC framework for aqueous transport and a LiCl‐doped Ecoflex layer that actively supplies mobile ions. This configuration enables high sensitivity to environmental humidity changes, enabling hydrovoltaic generation and humidity monitoring. As illustrated in Figure 1, the electricity generation mechanism of AEBC can be summarized as interfacial oxygen gradient construction, ambient moisture absorption, and synergistic ion transport: (1) The gradient distribution of oxygen‐containing functional groups between the GO/BC and CNT/BC regions establishes an asymmetric ion diffusion channel and an internal electric field. (2) Aqueous migration occurs as hygroscopic LiCl absorbs moisture exclusively from the soil, which is regulated by the confinement effect of the hydrophobic Ecoflex network, thereby suppressing lateral diffusion while enhancing vertical ion transport. (3) Cooperative ion transport is achieved through the combined contribution of Li^+^ generated from LiCl dissociation and H_3_O^+^ produced from water dissociation [19, 20]. These cations migrate directionally under the coupled driving forces of the water gradient and the internal electric field, ultimately resulting in effective charge separation and stable electrical output. The abundant surface hydroxyl groups of BC impart intrinsic hydrophilicity and provide sufficient moisture adsorption sites, while the nanofibrous network offers continuous capillary channels that facilitate rapid water transport and interfacial reactions [21, 22].

**FIGURE 1:**
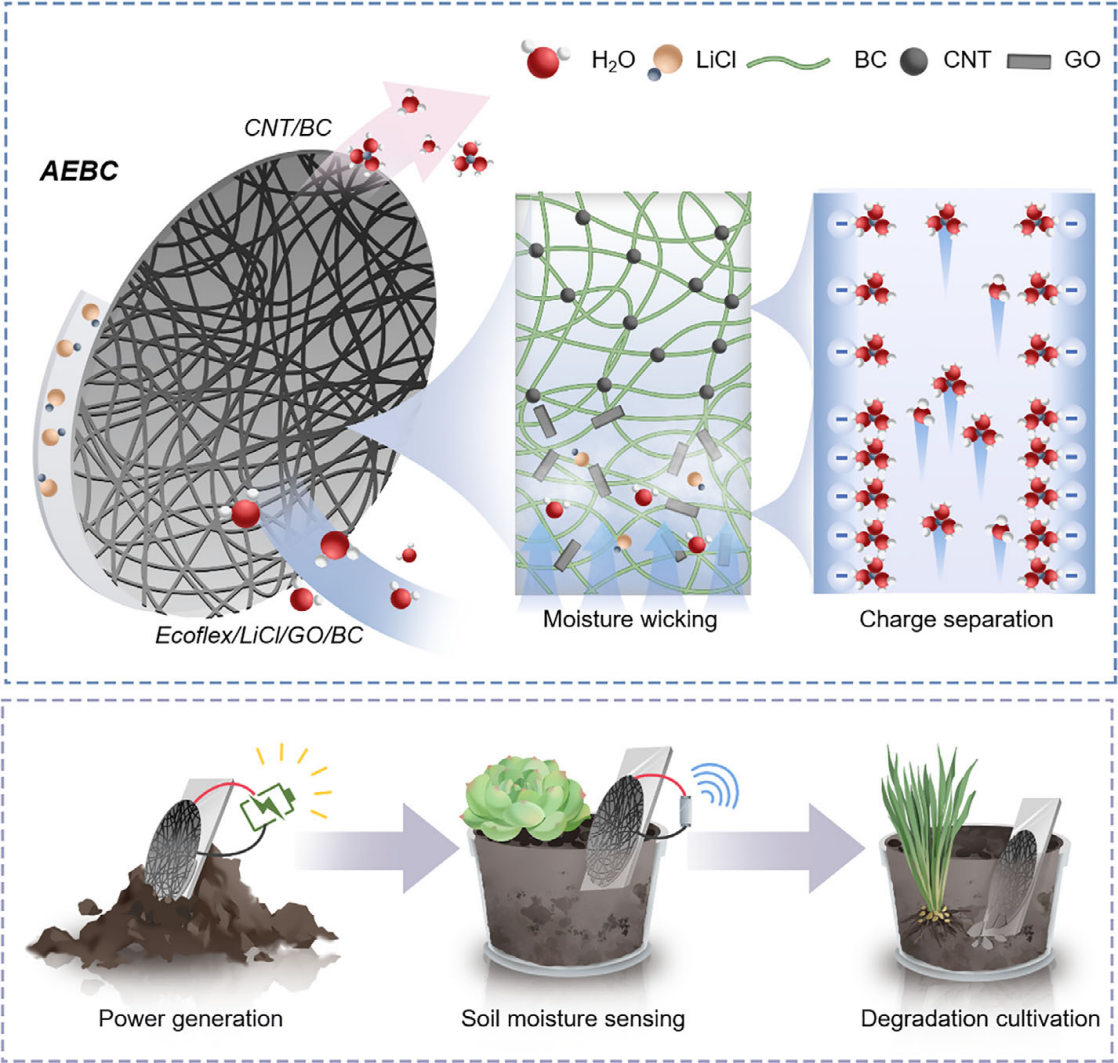
Operation mechanism of asymmetric encapsulated bacterial cellulose (AEBC) for self‐powered soil moisture monitoring and sustainable degradation.

As for AEBC, a combined strategy of microbial fermentation engineering and gravity‐induced assembly is proposed to fabricate an integrated heterogeneous structure (Figure 2A, Figure S1). Specifically, CNT and GO are separately added to the culture medium. During fermentation, a tilted culture condition is employed to enable in situ encapsulation of functional fillers by the growing BC, while gravity‐induced sedimentation leads to spatial differentiation of filler distribution. As a result, the carbon‐to‐oxygen ratio on the two sides of the composite membrane can be rationally altered to form a stable compositional gradient. Concentration optimization experiments reveal that excessively low functional material content limits the formation of conductive networks and interfacial polarization effects, whereas excessively high concentrations inhibit the growth of *Acetobacter xylinum* and disrupt the integrity of the fibrous network (Figure 2B, Figure S2). The most continuous conductive pathways and the lowest interfacial contact resistance can be achieved at a concentration of 1.2 wt.% (Figure S3). The successful fabrication of the integrated heterostructure is observable at scales from 100 to 10 µm. Meanwhile, EDS mapping analysis reveals a significant difference in the carbon‐to‐oxygen ratio between the opposite ends of AEBC (Figure 2C). According to the FTIR spectrum of BC/GO, the peaks at 3348, 2892, 1429, and 1061 cm^−1^ correspond to ─OH stretching vibration, C─H asymmetric stretching vibration, C─H asymmetric bending vibration, and C─O─C antisymmetric bridge oxygen stretching vibration, respectively; the peak at 1730 cm^−1^ is attributed to the C═O stretching vibration in GO (Figure 2D) [23, 24]. In contrast, the intensity of the hydroxyl peaks originally present in BC is significantly diminished in the CNT/BC composite. According to XPS analysis (Figure 2E), GO/BC exhibits higher oxygen content (37.62%) and a lower carbon‐to‐oxygen ratio (1.48), which enhances hydrophilicity and increases interfacial charge density, whereas CNT/BC shows an increased carbon‐to‐oxygen ratio (2.37). This difference in oxygen content leads to an asymmetric distribution of zeta potential and water affinity across AEBC (Figure 2F).

**FIGURE 2:**
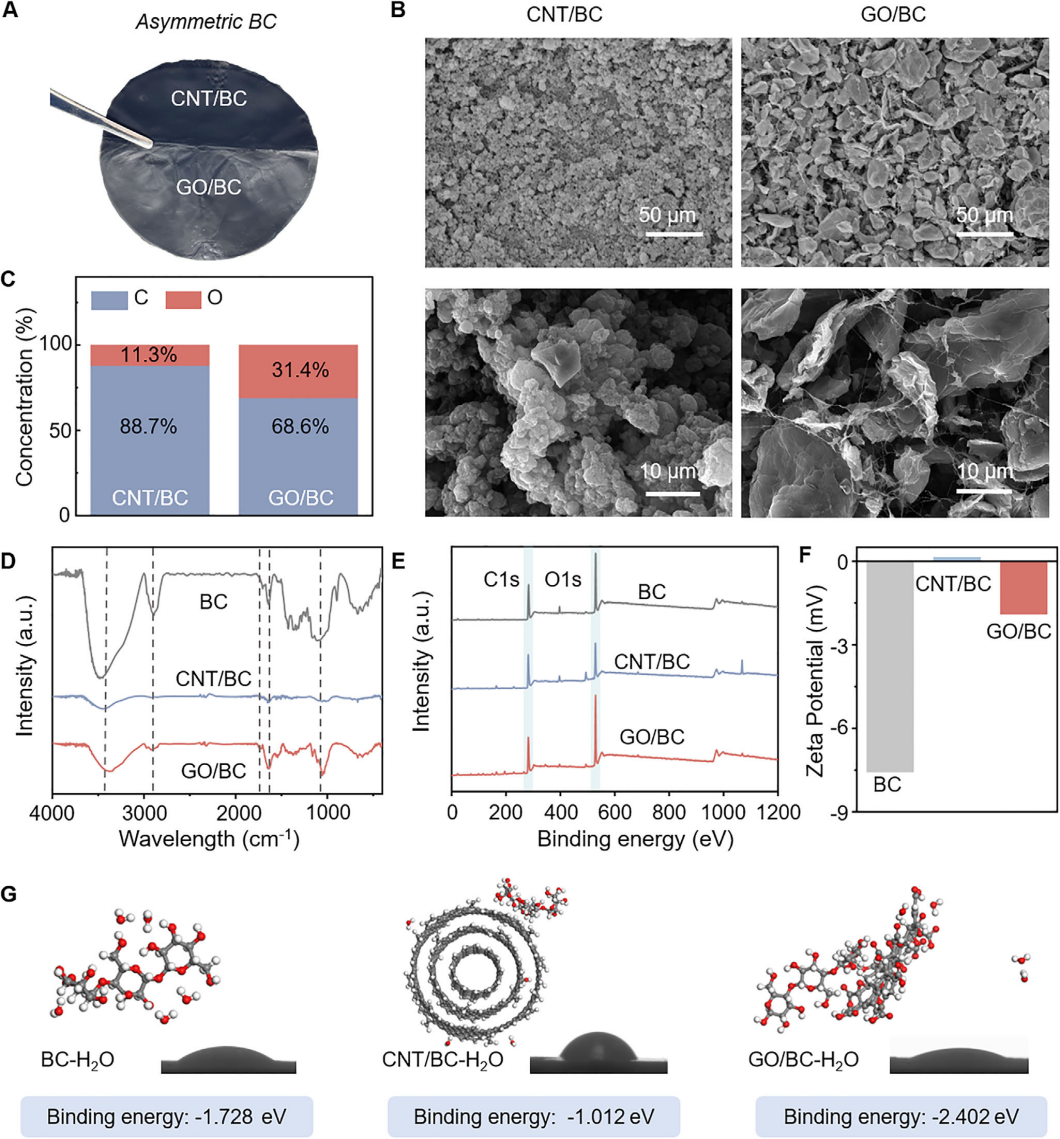
Construction and characterization. (A) Digital image of an asymmetric bacterial cellulose (BC) framework. (B) Scanning electron microscopy (SEM) image of carbon nanotube‐loaded BC (CNT/BC) and graphene oxide‐loaded BC (GO/BC). (C) Energy‐dispersive X‐ray spectroscopy (EDS) mapping of the C/O ratio for CNT/BC and GO/BC. (D) Fourier transform infrared (FTIR) spectra of BC, CNT/BC, and GO/BC. (E) X‐ray photoelectron spectroscopy (XPS) of BC, CNT/BC, and GO/BC. (F) Zeta potential of BC, CNT/BC, and GO/BC. (G) Comparison of water binding energy and water contact angle among BC, CNT/BC, and GO/BC.

The incorporation of GO weakens the original negative charge of BC but maintains an overall negative surface environment, while CNT introduction causes a reversal of zeta potential, resulting in a cross‐interface potential difference and an internal electric field for directional ion transport. According to water contact angle measurements, the distinct wetting behavior of water droplets on the material surface drives differential aqueous distribution, which facilitates the establishment of a potential gradient (Figure S4). Static binding energy calculations are performed using the Forcite module with the COMPASS III force field to evaluate the water adsorption affinity of each composite surface (Figure 2G). The results reveal that the GO/BC system exhibits the strongest water‐binding energy (−2.402 eV), which is attributed to the dense hydrogen bond network formed between oxygen‐containing functional groups on GO and hydroxyl groups of BC. In contrast, the CNT/BC system delivers a weaker binding energy (−1.012 eV), consistent with the hydrophobic graphitic surface of CNTs and their steric hindrance effect toward water molecule adsorption. This confirms that the gradient oxygen distribution establishes dual asymmetry of wettability and surface charge on the two sides of the device. The incorporation of GO and CNT partially disrupts the dense assembly of BC nanofibers and reduces the continuity of the load‐bearing network. In addition, heterogeneous filler‐fiber interfaces and local structural defects introduce stress‐concentration sites. Consequently, the functionalized BC composites exhibit lower mechanical strength than pristine BC [25]. Nevertheless, functional BC materials possess adequate mechanical strength to ensure stability and flexibility under long‑term operating conditions (Figures S5, S6).

### 2.2 Ion Transport and Hydrovoltaic Generation

Hydrovoltaic measurements of pristine BC, pure CNT/BC, pure GO/BC, and asymmetric BC with a deionized water droplet are conducted to simulate the asymmetric moisture‐absorbing environment in practical applications (Figure 3A). Pristine BC exhibits a near‐zero open‐circuit voltage under identical conditions, confirming that the hydrovoltaic output originates from the interfacial asymmetry introduced by the functional carbon materials rather than from the BC substrate itself. CNT/BC without enough oxygen functional groups exhibits weak water adsorption and a high energy barrier for dissociation, resulting in negligible ion generation and almost zero voltage output. Although GO/BC contains abundant oxygen‐containing functional groups to dissociate water molecules and produce ions, it only delivers a low voltage output. Its hydrophilic interface fails to create a clear hydrophilic‐hydrophobic discrepancy, thereby inhibiting the continuous buildup of ion concentration gradients. In contrast, the heterogeneous BC integrates hydrophobic CNT regions and hydrophilic GO regions within a continuous structure, forming a stable interfacial asymmetry. The directional ion transport originates from the synergistic coupling of interfacial electrostatic interactions and ion diffusion. The asymmetric distribution of oxygen‐containing functional groups modifies the surface‐charge states of the GO/BC and CNT/BC interfaces, thereby establishing an internal electrostatic driving force. Simultaneously, moisture uptake activates mobile ionic carriers and generates an ion‐activity difference across the asymmetric structure. The coupled electrostatic and diffusion driving forces collectively promote directional ion transport and generate the hydrovoltaic output [26, 27, 28, 29]. This configuration enables efficient water adsorption and dissociation at the GO side, while the hydrophobic CNT side restricts further water diffusion, establishing a sustained water activity gradient and an internal electric field for directional ion migration. Systematic optimization reveals that balanced conductive network construction, interfacial polarization, ion generation, and transport resistance can be achieved at 1.2 wt.% for both GO and CNT, enabling optimal device performance with a voltage output of 0.31 V and current output of 8.5 µA (Figures S7 and S8).

**FIGURE 3:**
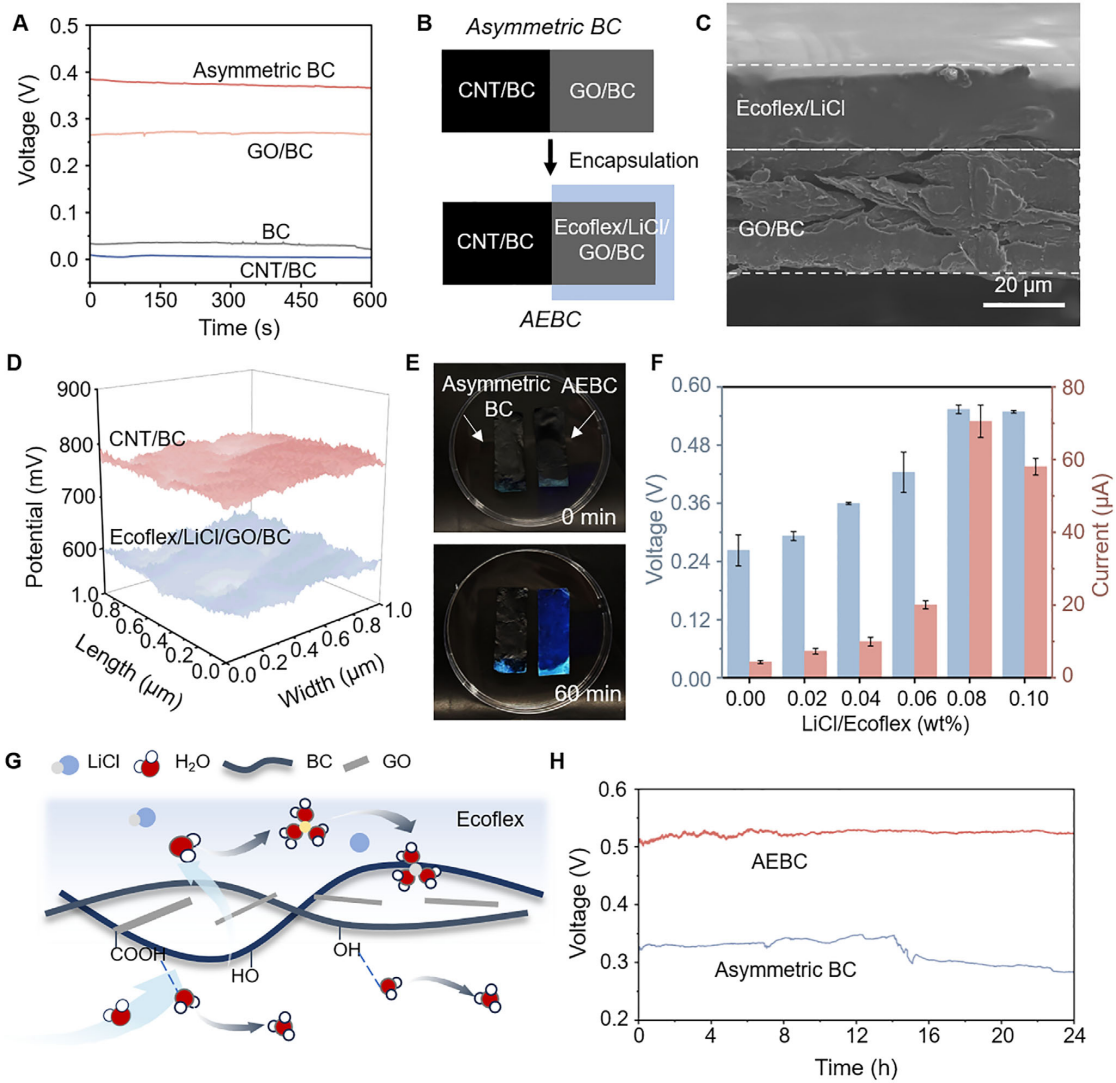
Hydrovoltaic operation mechanism. (A) Open‐circuit voltage of BC/CNT, BC/GO, and asymmetric BC under water dropping. (B) Schematic diagram of asymmetric BC and AEBC. (C) SEM image of cross‐sectional Ecoflex/LiCl/GO/BC layer. (D) Surface potential difference between the CNT/BC side and the Ecoflex/LiCl/GO/BC side of AEBC. (E) The fluorescein wicking performance after 60 min on AEBC and asymmetric BC. (F) Open‐circuit voltage and short‐circuit current of AEBC with different LiCl/Ecoflex mass ratios. (G) Schematic diagram of the hydrovoltaic generation mechanism of AEBC. (H) Open‐circuit voltage output of AEBC and asymmetric BC over 24 h of operation.

To further enhance moisture capture and ion supply, an Ecoflex/LiCl hygroscopic layer is introduced at the negative GO/BC side of asymmetric BC to fabricate AEBC (Figure 3B). SEM image reveals a clear interface between the hygroscopic layer and BC substrate (Figure 3C). The unique silicon element in the hygroscopic layer effectively confirms the designed multilayer structure, and the presence of Cl provides direct evidence for the distribution of the hygroscopic LiCl (Figure S9). The hydrophobic Ecoflex layer does not completely block moisture uptake but regulates the transport pathway of water. By suppressing uncontrolled lateral diffusion, the encapsulation layer confines moisture transport within the asymmetric structure. Meanwhile, the Ecoflex matrix physically restricts the migration and dissolution of the hygroscopic salt, thereby improving the retention of the internal ionic gradient during prolonged operation.

As shown in Figure 3D, there is a significant potential difference between the two sides of AEBC after incorporation of the hygroscopic salt. The hygroscopic LiCl continuously captures environmental moisture by reducing local vapor pressure, transforming the system from passive dropwise feeding to active moisture absorption. The dissociation of LiCl provides additional mobile ions, increasing the overall ion concentration. Meanwhile, the Ecoflex polymer network imposes spatial confinement that suppresses unordered water diffusion and promotes directional ion transport along the thickness direction, thereby maintaining a stable moisture gradient [30, 31]. Fluorescence diffusion experiments confirm that water diffusion is significantly reduced after encapsulation, indicating a transition from two‐dimensional diffusion to quasi‐one‐dimensional transport, which minimizes gradient dissipation (Figure 3E). Infrared images reveal pronounced thermal effects during moisture desorption and adsorption (Figure S10). Endothermic water desorption occurs with the temperature decreasing to 27.8°C. Exothermic water absorption occurs with the temperature increasing to 35.0°C. This temperature difference confirms the regulatory role of the encapsulation layer in directional water transport and provides a thermodynamic basis for the self‐sustaining humidity response of the device [32, 33, 34].

During hydrovoltaic operation, aqueous flow passively diffuses from the environment to the GO/BC end. Water molecules interact with the hydroxyl and carboxyl groups, undergoing protonation to form H_3_O^+^. Some water molecules further penetrate the Ecoflex/LiCl gel layer to induce the dissociation of LiCl into Li^+^ and Cl^−^, thereby forming stable Li(H_3_O)_n_^+^ [35, 36]. When the LiCl concentration is too low, it is difficult to establish an effective interfacial ion concentration gradient. Water uptake increases monotonically with LiCl loading without saturation, ruling out insufficient moisture at high concentrations (Figure S11). Voltage rises gradually at 0–0.04 wt.% due to limited ion activity, then accelerates at 0.06–0.08 wt.% as continuous transport pathways form with the enhancement of hygroscopicity. The current enhancement results from concurrent resistance reduction (Figure 3F). When the loading content exceeded 0.08 wt.%, the mobile ionic carriers gradually reached saturation. Although equilibrium water content tests revealed that the water absorption of the thin film continued to increase monotonically with concentration, the excessively increased water content diluted the effective ion concentration within the nanochannels [37, 38]. This optimal ratio not only ensures the stability of the gel network structure but also ensures rational ionic mobility (Figure 3G). By comparing different hygroscopic salts, it is found that Li^+^ features a small ionic radius, low electrostatic repulsion, and low ionic valence, which endows it with higher migration efficiency within nano‐confined channels and delivers comprehensively superior power output performance (Figure S12) [39]. Different saturated salt‑water solutions are prepared to establish varied atmospheric humidity environments (Figure S13). The power output performance of AEBC increases steadily under higher water‑vapor concentrations. The open‐circuit voltage measurements under forward and reverse bias conditions demonstrate that this strategy not only elevates the overall cation concentration but also substantially boosts the voltage output via directional ion migration (Figure S14).

During 24 h of continuous operation, the voltage of the unencapsulated asymmetric BC exhibits noticeable fluctuations and continuous decay, whereas AEBC with encapsulated Ecoflex/LiCl shows excellent hydrovoltaic stability, validating the critical role of the encapsulation layer in delaying salt loss and stabilizing ion supply (Figure 3H). As for hydrovoltaic output, AEBC with an average total thickness of only 35 µm achieves a volumetric power density of 211.37 µW/cm^3^ despite its ultrathin morphology (Figures S15 and S16). Compared with many Hydrovoltaic devices that employ thick‐film structures with hundreds of micrometers, AEBC demonstrates the advantage of the micro/nanofiber network in efficiently constructing ion transport channels and charge collection interfaces within a confined space (Table S1) [40, 41, 42, 43, 44, 45, 46]. AEBC exhibits outstanding output performance and operational stability. The electrical energy harvested by AEBC can also be stored in capacitors to meet intermittent operational demands (Figure S17). This indicates that AEBC is compatible with common energy storage components, laying the foundation for constructing fully self‐sustaining wireless monitoring systems. With the electrical signal amplification via series‐parallel connection, AEBC achieves a hydrovoltaic output of 4.3 V and 750 µA at nine connected units (Figure S18).

### 2.3 Self‐Powered Humidity Response and Wireless Monitoring System

Based on the confinement of LiCl within the Ecoflex matrix, moisture absorption becomes moderate and controllable, resulting in stable and distinguishable voltage responses under different soil humidity conditions (Figure 4A). Elevated soil humidity improves water activity to promote water dissociation at the GO interface and LiCl dissolution, thereby boosting H_3_O^+^ and Li^+^ concentrations. Meanwhile, the increased water content reduces local viscosity and ion transport resistance, which facilitates ion migration and gradually enhances the open‐circuit voltage. The matching relationship between soil moisture and hydrovoltaic output provides a solid physical basis for signal recognition (Figure S19) [47]. This correlation can be effectively captured and learned by the deep learning model. A confusion matrix is applied to systematically evaluate the reliability and generalization capability of the proposed deep learning model for humidity recognition (Figure 4B). It exhibits excellent classification performance across ten preset humidity levels, indicating that the model can accurately distinguish different soil humidity conditions from the hydrovoltaic signals. The constructed deep learning model effectively captures this nonlinear mapping, and its high classification accuracy of 98.6% indicates that the voltage signal contains sufficient information to distinguish humidity levels, confirming the reliability of AEBC as a self‐powered sensing unit. Loss and accuracy curves of the training and validation sets are provided in Figure S20. Both losses decline stably toward convergence, and the two sets exhibit consistent accuracy trends without persistent validation‑set deterioration or notable deviation, confirming sound model convergence and negligible overfitting.

**FIGURE 4:**
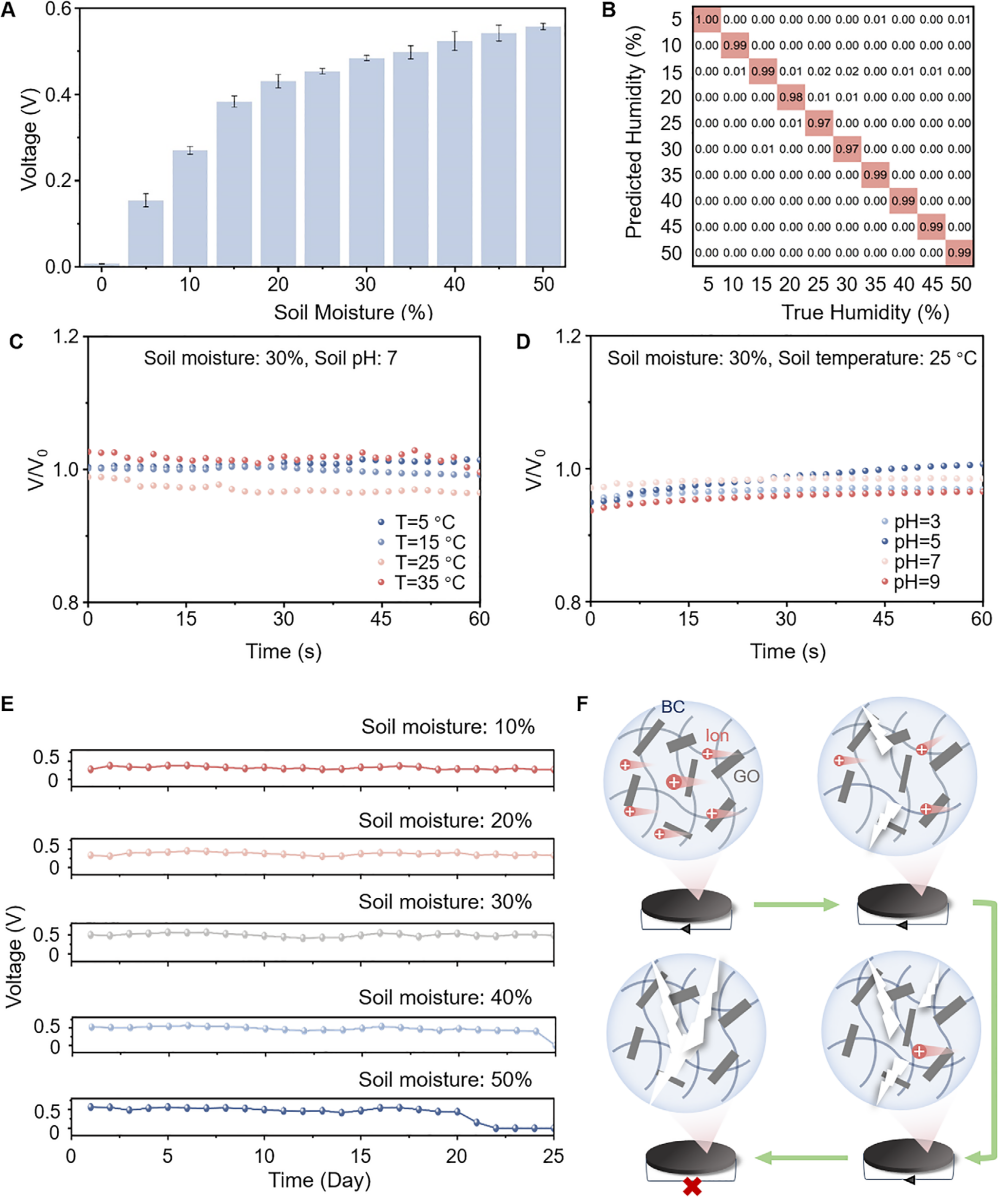
Self‐powered humidity monitoring. (A) Open‐circuit voltage of AEBC at different soil moisture levels. (B) Matching performance between the deep‐learning model‐predicted open‐circuit voltage for soil moisture response and the actual measured values. (C) Hydrovoltaic output stability at different soil temperatures. (D) Hydrovoltaic output stability at different soil pH values. (E) Long‐term hydrovoltaic output of AEBC at different soil moisture levels. (F) Schematic diagram of the degradation process of AEBC under soil conditions.

Soil, as a dynamically changing medium, exhibits significant fluctuations in temperature and pH due to seasonal variation, fertilization, and irrigation. AEBC exhibits temperature insensitivity because the hydrovoltaic driving force depends on evaporation‐induced ion migration. Within this range, the temperature‐enhanced ion mobility is counterbalanced by the strengthened water‐repelling effect of the hydrophobic CNT/BC side (Figure 4C). In addition, the Ecoflex encapsulation layer physically immobilizes LiCl, thereby preventing thermally accelerated ion loss. AEBC also maintains stable hydrovoltaic output over pH 3–9 (Figure 4D). The oxygen‐containing functional groups undergo reversible protonation and deprotonation depending on pH, stabilizing the zeta potential to preserve the driving force for directional ion migration. Meanwhile, the chemically inert Ecoflex encapsulation layer provides a physical barrier that isolates the LiCl ion source from H^+^/OH^−^ ions present in soil moisture, thereby suppressing hydrolysis and precipitation reactions. Mechanical testing demonstrates that hydrovoltaic performance remains stable under bending and cyclic deformation, indicating robust ion transport pathways supported by the flexible nanofibrous network, which maintains structural continuity during deformation (Figure S21).

Long‐term monitoring for 25 days under different humidity conditions is conducted to further evaluate the service life and output stability (Figure 4E). AEBC shows a clear response to humidity changes after being inserted into the soil. The output voltage stabilizes as the ion concentration gradually saturates. As the monitoring time extends, the degradation rate accelerates with increasing humidity. In high‐humidity soil environments, the output performance significantly declines until complete failure. This degradation is attributed to several coupled factors, including swelling‐induced disruption of the cellulose network in high humidity, redistribution of LiCl leading to weakened ion concentration gradients, and gradual loss of interfacial charge asymmetry (Figure 4F). During degradation, the Ecoflex LiCl layer swells and partially delaminates due to reduced interfacial adhesion, while the exposed GO/BC layer undergoes fragmentation under mechanical and microbial action in soil.

### 2.4 Sustainable Agricultural Monitoring

As shown in Figure 5A, a self‐powered wireless system is developed for soil moisture monitoring. The system integrates an analog‐to‐digital converter (ADC) for voltage acquisition, a microcontroller unit (MCU) for real‐time conversion of the voltage signal into humidity data, and a Bluetooth module for wireless data transmission (Figure 5B). To achieve real‐time humidity monitoring, we employ a preset calibration‐data‐based fitting formula as the conversion function on the MCU side. In line with the trend of digital information development, sensor data can be directly transmitted to smart terminals such as mobile phones. This process represents a direct transformation from physical signals to digital information, forming a closed loop from environmental humidity variation to signal output and feedback. The hydrovoltaic signal reaches equilibrium within minutes. By contrast, water molecules bound by cellulose hydroxyl groups and hygroscopic salts evaporate slowly from the film under high‑humidity conditions, which dissipates the internal ion gradient and thus leads to a prolonged signal‑recovery time (Figure S22). Taking a plant with an ideal growth humidity of 20%–30% as an example, when the soil is too dry (humidity below 20%), the mobile application interface displays a “sad face” icon with a “water shortage” alert (Figure 5C). When the humidity is within the moderate range (20%–30%), the interface switches to a “smiley face” icon, indicating appropriate moisture. When the humidity further increases to around 50%, the interface displays a “sad face” and issues an “excess water” warning. These results fully demonstrate the accurate identification and intuitive feedback capability of the system for soil humidity changes. Tests under varied atmospheric and soil humidities reveal distinct output signals for different soil‑moisture levels (Figure S23). Air humidity fluctuations cannot mask valid signals originating from soil‑moisture variation. Structurally, AEBC mainly draws water from the soil side. Unlike fully air‑exposed hydrovoltaic devices, continuous soil‑supplied water mitigates signal disturbances induced by short‑term, minor atmospheric‑humidity fluctuations. Even though Ca^2+^ and Mg^2+^ are ubiquitous within natural saline soils and can profoundly alter interfacial charge states and ion transport kinetics at soil interfaces, AEBC still delivers stable power outputs governed predominantly by soil moisture content across simulated saline soil concentrations (Figure S24). Such characteristics support dependable signal readout and monitoring operations [48].

**FIGURE 5:**
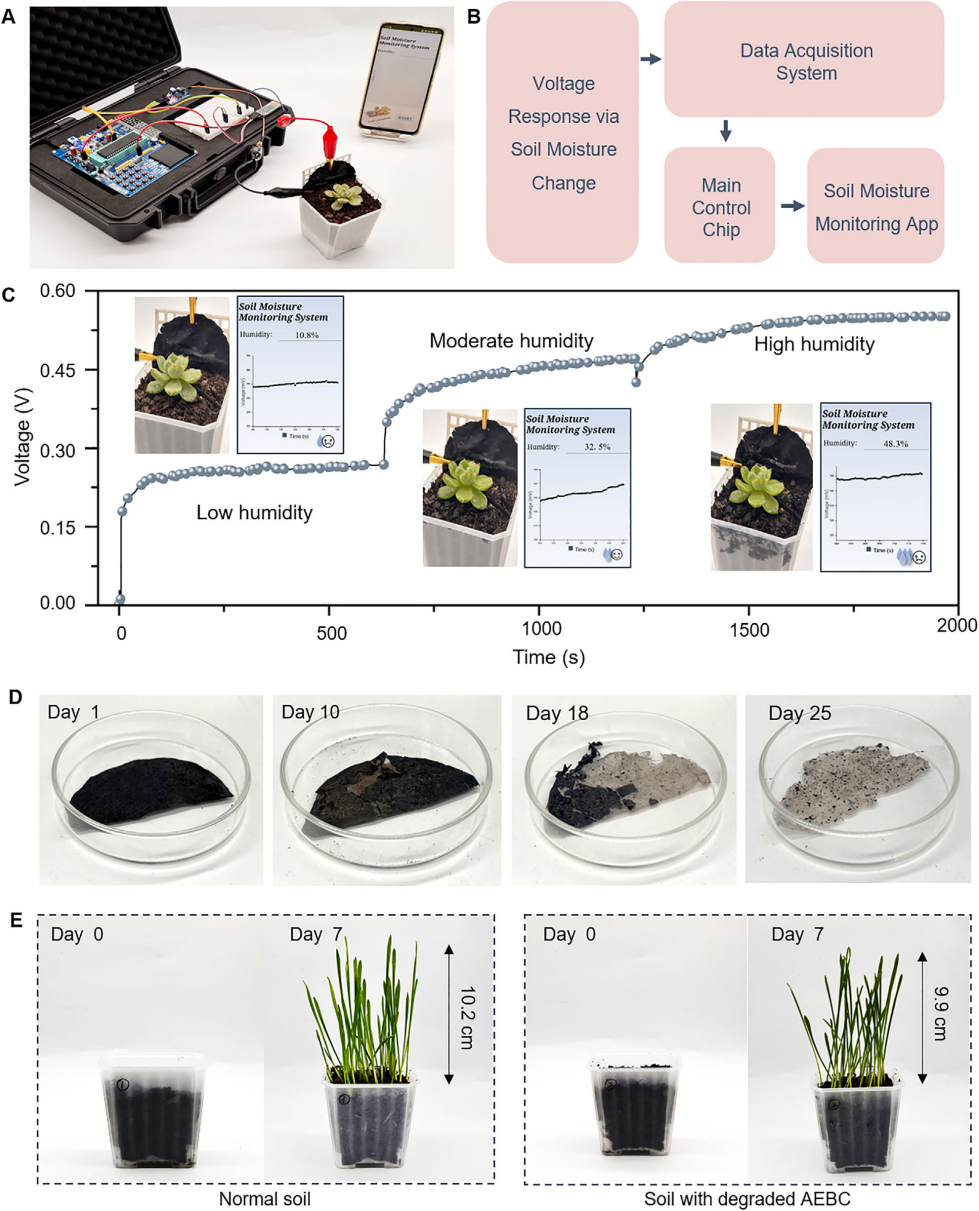
Prototype demo for sustainable agricultural monitoring. (A) The application setup of a self‐powered soil moisture monitoring system. (B) Overall workflow of the humidity sensing process. (C) Software demonstration during practical soil moisture monitoring. (D) Degradation process of AEBC during soil moisture monitoring. (E) The growth process of wheat in normal soil and in soil containing degraded AEBC fragments.

As mentioned above, although AEBC exhibits excellent moisture monitoring stability for over 20 days in soil environments, it inevitably undergoes gradual degradation and corrosion after its service life. This makes its ecological friendliness a critical criterion for practical deployment in sustainable agriculture. Initially, the Ecoflex/LiCl gel layer absorbs moisture, weakening its interfacial adhesion with the BC substrate and leading to partial delamination (Figure 5D). Subsequently, the exposed carbonaceous layer experiences mechanical abrasion from soil particles, gradually breaking into fragments. Finally, the BC nanofibrous network undergoes biodegradation by soil microorganisms, which hydrolyze the glycosidic bonds of cellulose into cellobiose and ultimately glucose. After long‐term operation, SEM images reveal delamination at the GO/BC interface, along with swelling and fracture of GO/BC fibers, indicating that water‐induced swelling and structural disruption are dominant failure pathways (Figure S25). EDS confirms chloride migration from the Ecoflex/LiCl layer throughout the film, directly diminishing the trans‐membrane ion gradient. KPFM shows increased surface potential on the GO/BC side due to cation adsorption shielding, reducing interfacial potential asymmetry (Figure S26). FTIR shows decreased ─OH intensity and disappearance of carboxyl ─C═O peaks, indicating loss of negative charge sites. Notably, weakened ─C─O─C peaks confirm cellulose chain scission (Figure S27).

To assess the potential impact of these degradation products from AEBC on the soil ecosystem, a parallel wheat planting experiment using normal soil and soil containing degraded AEBC fragments is conducted (Figure 5E). After 7 days of growth, wheat seedlings in both groups developed normally, with no statistically significant difference in plant height, indicating that the degradation products do not cause visible inhibition to either the soil environment or plant growth. Although Ecoflex is non‑degradable, its low loading content means it does not cause significant interference with seed growth. Despite the non‐biodegradable nature of Ecoflex, the minimal loading employed in this study poses no significant disturbance to seed germination or growth (Figure S28) [49]. Post‐harvest examination revealed that while some AEBC fragments adhered to the root surface, no abnormal root morphology or developmental defects are observed (Figure S29). The outstanding biosafety of AEBC degradation products stems from two aspects: biomass BC degrades into glucose, which serves as a universal carbon source for soil microbes, while the carbonaceous layer is reduced and fragmented into graphene‐like structures with low cytotoxicity [50]. These findings collectively demonstrate that AEBC fulfills the dual requirements of functional stability during operation and environmental safety after degradation. AEBC remains functional during service and becomes benign after disposal, achieving a complete functional loop of humidity indication, long‐term stable electricity generation, and natural degradation. Overall, AEBC provides a feasible pathway for sustainable self‐powered systems oriented toward green agricultural monitoring.

## 3 Conclusions

In summary, we have developed an asymmetric encapsulated bacterial cellulose (AEBC) device that addresses the critical challenges of self‐powered operation, long‐term stability, and environmental compatibility in soil moisture monitoring. The heterogeneous BC framework with gradient oxygen distribution establishes an internal electric field for directional ion transport. A LiCl‐doped Ecoflex hygroscopic layer actively captures moisture and provides a continuous ion supply. The selective encapsulation suppresses lateral water diffusion while maintaining vertical transport, thereby stabilizing the ionic concentration gradient. The synergistic combination of the above mechanisms transforms the hydrovoltaic process from a transient response into a self‐sustained non‐equilibrium driving system. AEBC achieves a volumetric power density of 211.37 µW/cm^3^ at an ultrathin thickness of 35 µm, outperforming many thick‐film hydrovoltaic devices. It maintains stable operation for over 20 days under varying soil conditions, with excellent tolerance to temperature fluctuation, pH variation, and mechanical deformation. The integration with a deep learning‐based wireless monitoring system enables accurate soil moisture sensing, demonstrating practical utility for precision agriculture. Furthermore, AEBC does not cause obvious adverse ecological impact on plant growth. Overall, this work establishes a complete loop of humidity indication, long‐term stable electricity generation, and natural degradation.

## 4 Method

### 4.1 Materials

Glucose, peptone, citric acid, disodium hydrogen phosphate, yeast extract, NaOH, LiCl, K_2_CO_3_, NaBr, KCl, NaCl, K_2_SO_4_, CaCl_2_ and MgCl_2_·6H_2_O were all purchased from Sinopharm Chemical Reagent Co., Ltd. Graphene oxide (purity 96%, 1–5 layers) and multi‐walled carbon nanotubes (purity 99%, length 8–14 µm) were supplied by Shenzhen Suiheng Graphene Technology Co., Ltd. Ecoflex Supersoft 00–50 silicone elastomer was obtained from Smooth‐On, Inc. (Pennsylvania, USA).

### 4.2 Sample Fabrication

#### 4.2.1 Asymmetric Bacterial Cellulose (BC) Fabrication

The Hestrin and Schramm (HS) medium was prepared with 5wt.% glucose, 0.5wt.% peptone, 0.5wt.% yeast extract, 0.6wt.% Na_2_HPO_4_, and 0.1wt.% citric acid monohydrate. A mixed solution of Acetobacter xylinus and HS medium was sprayed onto the surface of a clean culture dish. HS/CNT medium with the appropriate concentration was sprayed downward onto one half of the dish. The functional materials were immobilized by the wrapping action of BC fibers induced by gravity. This operation was repeated after 24 h. After the CNT/BC side had stabilized, HS/GO medium was sprayed onto the other side, and the above procedure was repeated to fabricate the integrated BC/CNT/GO device. The resulting hydrogel was subsequently boiled in 0.5 mol/L sodium hydroxide solution for 20 min and washed with deionized water to remove impurities to obtain asymmetric BC.

#### 4.2.2 Asymmetric Encapsulated Bacterial Cellulose (AEBC) Fabrication

LiCl powder at the corresponding concentration was uniformly mixed into uncured Ecoflex gel. The mixture was then coated onto the GO/BC side of asymmetric BC and dried in an oven at 45°C to obtain AEBC.

### 4.3 Characterization

The microscopic morphologies were observed by scanning electron microscope (Hitachi S‐4800, Sirion SEM) combined with energy‐dispersive X‐ray spectroscopy (EDS) mapping for elemental analysis. The chemical compositions were investigated through Fourier transform infrared (FTIR) spectroscopy (IRTracer 100 spectrometer, Shimadzu). The water contact angle test was carried out by an OCA15EC contact angle analyzer (Dataphysics, Germany). The electrical output was recorded by the electrometer (Keithley 7510). Infrared images were captured using an infrared thermal camera (FLIR T440). X‐ray photoelectron spectroscopy (XPS) was performed using a spectrometer (AXIS SUPRA, Shimadzu). Kelvin probe force microscopy (KPFM) measurements were conducted using an atomic force microscope (Bruker Dimension Icon). The zeta potential of the asymmetric cellulose film surface was measured using an Anton Paar Surpass instrument.

## Author Contributions

N.Z., J.L., and X.X. contributed equally to this article. K.L., W.X., and C.G. conceived and planned this research. N.Z., J.L., X.X., S.Y., B.C., S.W., and D.X. performed the experiments. C.G., D.X., and C.G. organized the data and wrote the manuscript. All authors discussed the results and approved the final version of the manuscript.

## Conflicts of Interest

The authors declare that there is no conflicts of interest regarding the publication of this article.

## Statistical Analysis

Data were analyzed using Origin 2025. Error bars generally represent the standard error of the mean, and data were obtained from at least three repeated measurements.

## Supporting information




**Supporting File**: advs78511‐sup‐0001‐SuppMat.docx.

## Data Availability

The data that support the findings of this study are available from the corresponding author upon reasonable request.
