# Supplementary material for: Gradient‐Oxidized Bacterial Cellulose for Self‐Sustained Soil Moisture Sensing
Source: Adv Sci (Weinh). 2026 Oct 27:e78511. Online ahead of print. doi: 10.1002/advs.78511 (PMC13711043; doi:10.1002/advs.78511)
Supplement: Supplementary file 1 [file ADVS-9999-e78511-s001.docx]

Supplementary Information

**Gradient-Oxidized Bacterial Cellulose for Self-Sustained Soil Moisture Sensing**

Nan Zhou ^1,†^, Jing Li ^1,†^, Xiang Xu ^1,†^, Senyuan Ye ^1^, Bin Chen ^1^, Shuang Wu ^5,6^, Duo Xu ^1,^*, Keshuai Liu ^1,^*, Chong Gao ^1,4,^*, Weilin Xu^1^, and Can Ge ^2,3,^*

^1^ State Key Laboratory of New Textile Materials and Advanced Processing, Wuhan Textile University, Wuhan 430200, China.

^2^ Department of Electronic Engineering and Shun Hing Institute of Advanced Engineering, The Chinese University of Hong Kong, Sha Tin, New Territories, Hong Kong 999077, China.

^3^ College of Textile and Clothing Engineering, Soochow University, Suzhou 215123, China.

^4^ College of Textile Science and Engineering, Zhejiang Sci-Tech University, Hangzhou 310018, China.

^5^ National Key Laboratory of Aerospace Chemical Power, Xiangyang 441003, China

^6^ Hubei Institute of Aerospace Chemical Technology, Xiangyang 441003, China

^†^ These authors contributed equally to this work

^*^ Corresponding author

Duo Xu ([dxu@wtu.edu.cn](mailto:dxu@wtu.edu.cn) );

Keshuai Liu ([ksliu@wtu.edu.cn](mailto:ksliu@wtu.edu.cn) );

Chong Gao ([gc2263506357@163.com](mailto:gc2263506357@163.com) );

Can Ge ([cange26@cuhk.edu.hk](mailto:cange26@cuhk.edu.hk));

This PDF file includes:

Supplementary Text 1-3

Fig. S1 to S29

**Supplementary Text 1: Soil Moisture Assessment**

Soil moisture was characterized by gravimetric water content, defined as the mass ratio of water to oven-dried soil (%). The moisture content is calculated as:

$$\text{Soil}\text{ }\text{Moisture}=\frac{m_{\text{water}}}{m_{\text{soil}}}\times100\%$$

Specifically, collected soil samples were dried at 65 °C to constant weight to obtain completely dry soil. Deionized water was then added at predetermined mass ratios, thoroughly mixed, and sealed for 24 h to ensure uniform water distribution. The soil moisture range covered 5% to 50%, encompassing agricultural soil conditions from arid to wet. A commercial soil moisture meter (model RS-MH-4G-C5-5+) was employed to ensure the accuracy and reproducibility of experimental conditions.

**Supplementary Text 2: Static Binding Energy Calculation**

All molecular simulation calculations were performed using the Forcite module in Materials Studio with the COMPASS III force field. Substrate models were constructed as follows: the BC substrate was built using cellulose Iβ disaccharide as the repeating unit, expanded along the a- and b-axes into a periodic supercell of approximately 50 Å × 50 Å, with a vacuum layer of no less than 15 Å along the c-axis to eliminate interlayer mirror interactions. The GO/BC substrate was prepared by grafting hydroxyl (-OH) and carboxyl (-COOH) groups onto the cellulose chain surface, with the same unit cell settings as the BC system. For the CNT/BC substrate, multi-walled carbon nanotubes were placed parallel to the cellulose chain surface, with the supercell dimensions adjusted accordingly to accommodate the CNT spatial scale. All three systems first underwent unit-cell-level structural pre-optimization, with atomic coordinates and cell parameters relaxed simultaneously.

During full-degree-of-freedom geometry optimization, electrostatic interactions were treated using Ewald summation (calculation accuracy: 1.0×10^-5^ kcal/mol), and van der Waals interactions were treated using Atom-based summation (cutoff distance: 15.5 Å, with long-range correction enabled). Convergence criteria were set as follows: energy threshold of 1.0×10^-4^ kcal/mol, force threshold of 0.005 kcal/(mol·Å), and displacement threshold of 1.0×10^-5^ Å. For binding energy calculations, a cluster of five water molecules was placed at the most stable adsorption region on the optimized substrate surface, with all substrate atomic coordinates fixed and only the water cluster subjected to geometry optimization. The binding energy was defined as:

$$E_{\text{binding}}=E_{\text{total}}-E_{\text{substrate}}-E_{\text{water}}$$

Where $E_{\text{total}}$ is the total energy of the composite system, $E_{\text{substrate}}$ is the single-point energy of the substrate without water, and $E_{\text{water}}$ is the single-point energy of the five isolated water molecule cluster. Both$E_{\text{substrate}}$and $E_{\text{water}}$were obtained by directly extracting atomic coordinates from the optimized composite configuration for single-point energy calculations, without secondary geometry relaxation of the substrate or water cluster. Therefore, the binding energy reflects the adsorption interaction under fixed conformation and does not include substrate conformational rearrangement energy. This work reports the total binding energy between the composite material and five water molecules. The binding energy results in this work are primarily used for comparing relative trends in interaction strength, rather than interpreting absolute energy values.

**Supplementary Text 3: Deep Learning Classification**

Soil moisture classification was performed using a convolutional neural network (CNN)-based classification model. Voltage signals were categorized into 10 soil moisture levels (5%-50%, 5% step). The dataset comprised 2,000 samples (200 per class) collected under RH 70-80% across different soil moisture environments, from multiple independent devices and different fabrication batches. To avoid temporal data leakage, the dataset was split by acquisition batch into 80:10:10 for training, validation, and test sets, ensuring that continuous time-series samples from the same group were not distributed across different datasets. The loss and accuracy curves for the training and validation sets demonstrate that both losses decreased steadily and eventually converged during training, with accuracy trends remaining essentially consistent. Validation set performance did not exhibit persistent decline or significant divergence from the training set, indicating good convergence and no obvious overfitting.

**Supplementary Figures**

Fig. S1. Schematic diagram of the preparation process of AEBC.

Fig. S2. Photographs of composite BC with different CNT/HS and GO/HS concentrations.

Fig. S3. Resistance of composite BC with different CNT/HS and GO/HS concentrations.

Fig. S4. Water contact angle measurement of BC, CNT/BC, GO/BC, Ecoflex/LiCl/GO/BC.

Fig. S5. Photographs of Asymmetric BC under folding and twisting states.

Fig. S6. Stress-strain curves of BC, CNT/BC, GO/BC, and asymmetric BC.

Fig. S7. Hydrovoltaic output of asymmetric BC with fixed 1.2 wt% CNT/BC and different GO/HS concentrations.

Fig. S8. Hydrovoltaic output of asymmetric BC with fixed 1.2 wt% GO/BC and different CNT/HS concentrations.

Fig. S9. EDS elemental mapping of Ecoflex/LiCl/GO/BC.

Fig. S10. Infrared thermal images of AEBC showing temperature difference between the dry (left) and hygroscopic (right) regions under dark and radiation conditions.

Fig. S11. Water content of Ecoflex/LiCl composite film over 12 h at 50 % soil moisture.

Fig. S12. Hydrovoltaic output of AEBC with different hygroscopic‑salt loadings.

Fig. S13. (A) Digital image of hygroscopic‑salt solutions prepared for different humidity conditions, (B) The hydrovoltaic generation performance of AEBC under various humidity conditions.

Fig. S14. Open-circuit voltage of the AEBC under forward and reverse connection conditions.

Fig. S15. Thickness measurement of AEBC

Fig. S16. Hydrovoltaic output of AEBC under different external resistances.

Fig. S17. Capacitor charging process via AEBC.

Fig. S18. Hydrovoltaic output of amplified AEBC.

Fig. S19. The matching relationship between the voltage output of AEBC and soil moisture.

Fig. S20. The variation in (A) loss and (B) accuracy during training with increasing epochs.

Fig. S21. Performance stability of AEBC under different mechanical bending states.

Fig. S22. Output-voltage response under an air humidity of 70% with different soil moisture conditions.

Fig. S23. Voltage output of AEBC at different atmospheric relative humidity (50, 60, 70, 80, 90 %) and soil moisture humidity (15, 25, 50 %).

Fig. S24. (A) Voltage output of AEBC within mixed CaCl_2_/MgCl_2_ salts at soil moisture of 30% with air humidity of 70%. (B) Voltage output of AEBC at varied soil moisture and air humidity conditions under 150 mM mixed CaCl_2_/MgCl_2_ salts.

Fig. S25. Surface SEM images of GO/BC and CNT/BC under different operating cycles and their corresponding EDS mapping of the Cl element.

Fig. S26. Surface potential difference between the CNT/BC side and the Ecoflex/LiCl/GO/BC side of AEBC after 20 days of operation.

Fig. S27. FTIR spectra acquired from the pristine GO/BC surface of AEBC and following 25 days of degradation.

Fig. S28. Digital images showing the effects of the presence or absence of Ecoflex in soil on wheat growth.

Fig. S29. Roots of grown wheat seedlings in normal soil versus those in soil with degraded AEBC.

**Tab. S1.** Performance comparison of AEBC and recent hydrovoltaic devices.

| **Material system** | **Current**  **(μA)** | **Volumetric power density (μW·cm^-3^)** | **Long-term stability** | **Ref** |
| --- | --- | --- | --- | --- |
| Wood/GO | 43 | 29 | 220 h | [1] |
| PPC/Li-PMC | ＞600 | 53 | 36 h | [2] |
| PU/PNa/PSS | 27.5 | 52.5 | 330 h | [3] |
| CP/PSS/CBNP | 96 | 98.04 | 120 h | [4] |
| CF/PAM/Gly/NaCl | 15 | 3.7 | 140 h | [5] |
| PA-AM/LiCl/Gly | 14.5 | 7.7 | 67 h | [6] |
| Carbon Fabric/PVA-NaCl | 7 | 70 | 150 h | [7] |
| **AEBC** | **58.8** | **211.3** | **20 day** | **This work** |

**Reference**

1.	C. Guo; H. Tang; D. Kong; Q. Chen; X. Wu; F. Fan; X. Zhao; R. Ding; W. Zhong; D. Zhao, *Energy Environ. Sci.* **2026,** *19*, 230-240.

2.	G. Huang; J. Liu; H. Zhang; W. Zhang; Y. Deng; J. Sha, *Chem. Eng. J.* **2025,** *504*, 158878.

3.	X. Wen; Z. Sun; M. S. Kim; Y. Cho; H. Wang; J. Yin; L. Zhang; I. K. Oh; L. Wang; I. D. Kim; X. Qin, *Adv. Funct. Mater.* **2026,** *36* (39), e11843.

4.	Y. Yuan; J. Ma; B. Li; S. Liu; Y. Li; Y. Zhang; X. Nian; K. T. Lin; C. Guo; H. Lin; B. Jia, *Small* **2025,** *21* (50), e09673.

5.	X. Wen; Z. Sun; Y. Cho; M. S. Kim; K. Qin; Q. Zhou; C. Zhang; L. Wang; I. D. Kim; X. Qin, *Adv. Funct. Mater.* **2025,** *35* (51), e06700.

6.	Z. Sun; X. Wen; J. Kim; K. Zhao; Q. Zhou; M. Mahato; V. H. Nguyen; Y. Yang; L. Wang; I. K. Oh, *Adv. Funct. Mater.* **2025,** *36* (7), e18814.

7.	Y. Zhang; S. Guo; Z. G. Yu; H. Qu; W. Sun; J. Yang; L. Suresh; X. Zhang; J. J. Koh; S. C. Tan, *Adv. Mater.* **2022,** *34*, 2201228.
